# *omp*A Sequencing and Multilocus Sequence Typing of Lymphogranuloma Venereum Cases in Buenos Aires Reveal New *Chlamydia trachomatis* Genotypes

**DOI:** 10.3390/microorganisms12030587

**Published:** 2024-03-15

**Authors:** Karina Andrea Büttner, Andrea Carolina Entrocassi, María Lucía Gallo Vaulet, Deysi López Aquino, Dolores Caffarena, Luciana La Rosa, Laura Svidler López, Osvaldo Degregorio, Björn Herrmann, Marcelo Rodríguez Fermepin

**Affiliations:** 1 Universidad de Buenos Aires, Facultad de Farmacia y Bioquímica, Microbiología Clínica, Buenos Aires C1113AAD, Argentina; karina.buttner@gmail.com (K.A.B.); carolinaentrocassi@gmail.com (A.C.E.); lgvaulet@ffyb.uba.ar (M.L.G.V.); 2 Universidad de Buenos Aires, Instituto de Investigación en Fisiopatología y Bioquímica Clínica (INFIBIOC), Buenos Aires C1113AAD, Argentina; 3 División Cirugía, Hospital Fernández, Buenos Aires C1425AGP, Argentina; lopezdeysi20@gmail.com (D.L.A.); lausvidlerlopez@gmail.com (L.S.L.); 4 Centro Privado de Cirugía y Coloproctología, Buenos Aires C1060ABB, Argentina; dolorescaffarena@gmail.com (D.C.); lucianalarosa@gmail.com (L.L.R.); 5 Universidad de Buenos Aires, Ciencias Veterinarias, Salud Pública, Buenos Aires C1427CWO, Argentina; odegre@fvet.uba.ar; 6 Department of Clinical Microbiology, Uppsala University Hospital, 751 85 Uppsala, Sweden; bjorn.herrmann@medsci.uu.se; 7 Section of Clinical Microbiology, Department of Medical Sciences, Uppsala University, 751 85 Uppsala, Sweden

**Keywords:** *Chlamydia trachomatis*, Lymphogranuloma venereum, sexually transmitted infections

## Abstract

Since the Lymphogranuloma venereum (LGV) outbreak was first described in Buenos Aires in 2017, the detected strains presented peculiar characteristics. Our goal was to increase the understanding of the strains involved in the LGV outbreak in Argentina. We characterized the *omp*A gene sequences, using Sanger sequencing, of 88 LGV strains from 239 symptomatic patients in Buenos Aires enrolled between 2017 and 2019, and selected 20 *C. trachomatis* strains for further characterization using Multilocus Sequence Typing (MLST). Following the *omp*A gene analysis of the 88 LGV strains, we detected 43% L2b, 31% L1-like, and 26% L2. Among the 38 L2b samples analyzed, there were 7 distinct sequences, 3 of them not previously reported (L2bv12, L2bv13, and L2bv14). Additionally, we detected a strain with a new mutation (AM884176.1:g.59122A>T) found in the position defining L2 or L2b, proposed as L2i. Using MLST, five different sequence types (STs) were detected, including the ST2 (corresponding to the L1-like strains) and a new one (ST60). ST58 was associated with the concomitant presence of another STI and HIV. A high genetic diversity in *C. trachomatis* LGV strains in Argentina was observed in a short period of time, with a relatively low number of samples from a limited geographical area.

## 1. Introduction

*Chlamydia trachomatis* is the most prevalent bacterial sexually transmitted infection (STI) worldwide. In 2020, the World Health Organization (WHO) estimated that approximately 128 million new *C. trachomatis* sexually transmitted infections occurred globally [1]. In Argentina, there are no general prevalence data, as *C. trachomatis* detection has not been incorporated into routine diagnostics [2]. A prevalence study in Buenos Aires in 2007 revealed an overall infection rate of 1.9% among adults [3]. Another study conducted in the province of Buenos Aires between 2016 and 2017, focusing on pregnant women, detected *C. trachomatis* in 18% of them [4]. These isolated studies emphasize the lack of comprehensive nationwide prevalence data for *C. trachomatis* in Argentina, highlighting the need for broad-scale investigations to grasp its prevalence and impact across the general population.

Since 2003, LGV outbreaks have been reported in men who have sex with men (MSM), most of them infected with the Human Immunodeficiency Virus (HIV), in different countries around the world [5,6,7].

In 2017, the first LGV cases detected in Argentina were similar to those reported in Europe since 2003, mostly HIV-infected male patients who had sex with men. Most cases presented symptomatic manifestations, demonstrating varying degrees of proctitis (mild, moderate, and severe), with associated clinical features such as straining, tenesmus, urgency for bowel movements, and mucous or purulent discharge. In addition to HIV co-infection, 40% of them were diagnosed with at least another associated STI [8,9].

In Argentina, considering the cases reported to the Ministry of Health from the Instituto Nacional de Microbiología Carlos G. Malbran [2] and those contributed by the Laboratorio de Clamidias, there is an average of 60 LGV cases reported per year, which results in an incidence of 20 cases per million inhabitants in the city of Buenos Aires. This incidence value needs to be regarded as the lowest, since, as of today, no other laboratories can identify LGV from non-LGV cases of *C. trachomatis* and inform them to the Argentinian health surveillance system. Even considering that, this value is 50% higher than the reported LGV rate in Europe, estimated to be 13 cases per million inhabitants [10].

Since the first description of the LGV outbreak in Buenos Aires, the detected strains of *C. trachomatis* showed unique characteristics. About half of the studied strains belonged to the L2b genotype, 20% to the L2 genotype, and one-third to the L1 genotype, which was absent in the European outbreak [9,11,12,13].

*C. trachomatis* typing is traditionally based on the sequencing of the highly variable *omp*A gene [14,15]. Genotyping is important for the identification of LGV strains, since they are more invasive and require extended antibiotic treatment compared to strains with other *omp*A genotypes [16,17]. Despite this, studying only a single region of the genome is insufficient to comprehend the complexity of genomic diversity in LGV outbreak samples at both global and local levels. To overcome this limitation, Multilocus Sequence Typing (MLST) offers a comprehensive approach. MLST involves PCR amplification and the subsequent DNA sequencing of multiple *loci* within the genome. Three MLST schemes have been proposed for *C. trachomatis* typing. Two of them are based on the analysis of constitutively expressed housekeeping genes, providing resolution comparable to complete *omp*A gene sequencing and making them suitable for evolutionary studies, whilst not as good for outbreak or clinical–epidemiological studies [18,19]. In 2007, Klint et al. introduced a third system, designed for short-term epidemiological studies. The MLST scheme proposed by Klint et al. has undergone slight modifications to optimize processing, resulting in only a one percent reduction in the discriminatory capacity [20]. The current configuration is accessible through the PubMLST//*Chlamydiales* database (https://pubmlst.org/organisms/chlamydiales-spp, accessed on 24 November 2023). This scheme analyzes five highly variable yet stable genomic *loci* (*hctB*, CT058, CT144, CT172, and *pbpB*), offering up to five times the resolution power of *ompA* gene sequencing [21].

Considering the scarcity of data regarding the prevalence of *C. trachomatis* in Argentina and Latin America, and the unique aspect of the 2017 LGV outbreak in Buenos Aires, characterized by the presence of an L1-like strain in one-third of the diagnosed cases, there exists a significant gap in understanding the epidemiology of LGV in the region. Furthermore, unlike in Europe, there is a notable lack of epidemiological and microbiological data on lymphogranuloma venereum in South America. This study aims to address this gap by investigating the epidemiology and its correlations with the clinical presentation of LGV in Argentina. To achieve this, we have applied *omp*A sequencing and MLST.

## 2. Materials and Methods

A total of 239 anorectal swabs were obtained from patients with symptoms compatible with proctitis between September 2017 and August 2019. Adult patients were prospectively included from a public hospital (Hospital Juan A. Fernández, Buenos Aires, Argentina) and a private health center (Centro Privado de Cirugía y Coloproctología, Buenos Aires, Argentina) in Buenos Aires, Argentina. Inclusion criteria encompassed individuals aged 18 years and above who provided written informed consent, seeking consultation for anorectal symptoms including rectal tenesmus, anal discharge, bowel urgency, and proctalgia, and who had not taken antimicrobial drugs in the previous month. Exclusion criteria included individuals under 18 years of age, those with a history of pelvic radiotherapy, and those previously diagnosed with inflammatory bowel disease. No patient was referred by another participant in the study. Inflammatory bowel disease and pelvic radiotherapy patients were excluded. Patients were treated according to current STI guidelines [22,23].

A thorough anamnesis was taken, and anal samples were collected and analyzed at the Laboratorio de Clamidias (Facultad de Farmacia y Bioquímica, Universidad de Buenos Aires, Buenos Aires, Argentina). Briefly, total DNA was isolated from clinical samples using Quick-DNA MiniPrep (Zymo Research Corporation, Irvine, CA, USA). *C. trachomatis* was tested using a real-time PCR targeting the cryptic plasmid (CHLAMYDIA tr. Q-PCR Alert kit; ELITech Molecular Diagnostics, Bothell, WA, USA). All real-time PCR-positive samples were genotyped by the PCR-RFLP of the *omp*A gene, as previously described. A nested PCR targeting the *ompA* gene was initially performed. The first PCR round generated a 1.1 kb DNA fragment of the *ompA* gene [24]. Then, a nested PCR was conducted, yielding a DNA fragment spanning from positions c.55 to c.1067 within the *omp*A gene of the *C. trachomatis* DNA sequence L2b:JN795427.1. RFLP analysis of the nested PCR-positive samples followed the protocol described by Sayada et al., with minor adjustments [25]. Briefly, 10 µL of the positive nested PCR product underwent digestion with 2.5 U *Alu*I (Promega Corporation, Madison, WI, USA) following the manufacturer’s guidelines. Subsequently, samples were subjected to 10% polyacrylamide gel electrophoresis, stained with Gel Red (GENBIOTECH SRL, Gel Red 10,000 X in 500 µL water per the manufacturer’s instructions) for 15 min, and photographed under UV light [24]. When necessary, additional analyses were performed using a second enzyme (*Hinf*I, CfoI, or a combination of *EcoR*I and *Dde*I). Genotypes were confirmed by sequencing the nested PCR product using capillary electrophoresis sequencing (Sanger sequencing), which covers 86% of the *ompA* gene (Macrogen, Inc., Seoul, Republic of Korea). These sequences were analyzed against the NIH databases using the BLAST tool (https://blast.ncbi.nlm.nih.gov/Blast.cgi, accessed on 24 November 2023). These sequences have been deposited in GenBank (MN548736.1 to MN548759.1, MN537150.1 to MN537152.1, and MN563608.1 to MN563615.1).

Based on the *omp*A typing results, 20 *C. trachomatis* samples were selected for high-resolution typing by MLST according to the Uppsala scheme (https://pubmlst.org/static/organisms/chlamydiales-spp/Protocol_MLST_C_trachomatis_Uppsala.pdf, accessed on 24 November 2023) [20]. Selected samples were subjected to PCR amplification and sequencing of the five target regions (CT144, CT172, CT058, and regions of the *pbp*B and *hct*B gene) and the *omp*A gene. The obtained sequences were analyzed using BioEdit software (version 5.0.9). An allele number was assigned by comparing the sequences at each *locus* with all corresponding known alleles available in the *C. trachomatis* PubMLST//*Chlamydiales* database (https://pubmlst.org/).

Allele profiles based on the five genetic regions generated sequence types (STs). The obtained sequences were deposited in the PubMLST database (Acc. No. 5059–5074). We performed two GrapeTree analyses using the resources provided in the PubMLST//*Chlamydiales* database (https://pubmlst.org/bigsdb?db=pubmlst_chlamydiales_isolates, accessed on 24 November 2023).

Statistical analysis was performed using InfoStat version 2020 software. The Fisher exact test or the Irwin Fischer test were used for categorical variable analysis. Statistical significance was defined as *p* < 0.05.

## 3. Results

### 3.1. Patient Characteristics and ompA Gene Variability in C. trachomatis Strains

The study included 239 patients aged 18 years and older (18–70, mean 33), comprising 9 cisgender women, 8 transgender women, and 222 cisgender men. All reported engaging in unprotected sexual intercourse. The overall HIV positivity rate among the participants was 63%. *C. trachomatis* was detected by PCR in 107 (45%) of the 239 patients, 1 cisgender woman, 2 transgender women, and 104 cisgender men, all of them MSM. The mean age of *C. trachomatis*-infected patients was 34 years (18–59 years), and 78% were HIV-positive and undergoing treatment. PCR-RFLP genotyping revealed that 88 out of 107 (82%) belonged to an LGV genotype. Of the LGV-positive patients, 87 were MSM and 1 was a transgender woman. The mean age was 34 years (21–59 years), and 86% were HIV-positive and receiving treatment.

Successful *omp*A sequencing was obtained for 104 (97%) positive samples, of which 85% (n = 88) were LGV and 15% (n = 16) were non-LGV genotypes. Regarding LGV genotypes, close to half were identified as the *ompA* genotype L2b (n = 38, 43%), whereas one-fourth of the samples were the *omp*A genotype L2 (n = 23, 26%) and one-third were similar to the L1 genotype (n = 27, 31%). The 27 L1 strains identified by PCR-RFLP were identical in the *omp*A sequence analysis and exhibited up to 10 mutations compared to the L1/440 based on the length of the obtained sequence (DQ064294.1: c.268G>A, c.348T>A, c.462C>T, c.471G>A, c.474A>G, c.477C>T, c.594C>T, c.931A>G, c.1017C>T, and c.1020A>C.). Hence, we named it as L1-like. Moreover, one of these L1-like strains had an additional *omp*A mutation (DQ064294.1:c.507A>C). The mutation in this L1-like strain (at position 931 in the L1/440 reference sequence DQ064294.1:c.931A>G) has not been previously described.

Among the 23 L2 strains, 22 were identical to the reference sequence AM884176.1, and 1 had a mutation at position AM884176.1: g.59122A>T. Notably, this single mutation in the *ompA* gene was located precisely at position 485, which is the position that distinguishes L2 from L2b. Consequently, this particular strain cannot be classified according to the convention used for L2/L2b [26]. We have initially designated this variation, which has not been previously documented in the databases, as “L2nv (new variant)” (additional information in the Supplementary Table S1). The distribution of the LGV L2b *omp*A genotype designations in our sample set is summarized in Table 1. Among the 38 samples analyzed, 29 (76%) were classified as the L2b genotype. Notable variations were observed, with a subset of samples falling into distinct *omp*A genotype designations, such as L2bv1 (four, 10%), L2bv5 (one, 2.6%), and L2bv11 (one, 2.6%). Three different strains (7.9%) lacked *omp*A designations in the referenced study by Helena M. B. Seth-Smith et al. (2021) [27] and were not found in the BLAST database. Following the nomenclature used in that study, we named them L2bv12, L2bv13, and L2bv14 [27]. Noteworthy, all the found mutations generated conservative amino acid substitutions, except for one demonstrating semi-conservative alteration. All mutations, except one, were confined within the variable domains of the *omp*A gene [28,29,30,31,32].

### 3.2. Novel C. trachomatis MLST Sequence Type and Uncommon Genotype Prevalence

For the MLST analysis, we selected a total of 20 samples, with 10 identified as the L2b genotype, 6 as L1-like, and 4 as L2. Within the L2b subset, we analyzed two L2b types, three L2bv1 types, one L2bv5 type, one L2bv11 type, one L2bv12 type, one L2bv13 type, and one L2bv14 type. Within L1-like samples, we included the one featuring the additional mutation. Among the L2 samples, three showed identical sequences to the reference sequence AM884176.1, while one exhibited a mutation at position AM884176.1: g.59122A>T.

The analysis resulted in high-quality sequencing results for 16 out of the 20 samples (Table 2). Unfortunately, four samples corresponding to the L2b *omp*A genotype could not be analyzed due to insufficient amplification of the target genes (two L2bv1, one L2bv11, and one L2bv13). We identified the following five distinct sequence types according to the Uppsala scheme: ST2, ST58, ST141, ST143, and a novel ST designated as number 60 (Table 2).

Upon closer examination of the five identified STs, three of them corresponded to previously well-known sequence types (ST58, ST141, and ST143). The L1-like samples, identified as genotype ST2, represent the only ST2 sequences with DNA sequences available in the PubMLST database. Additionally, an unprecedented combination of allele *loci* was observed, generating a novel ST (CT058 13; CT144 17; CT172 13; *hct*B 18; *pbp*B 28) designated as ST60, recorded under the accession number 5067 in the PubMLST database.

According to the *omp*A analysis, the samples classified as belonging to the L2b genotype were identified as either MLST ST58 or ST143, both of which have been previously associated with L2 and L2b strains. Among the four L2 samples, one exhibited ST58, one exhibited ST141 (both of which were formerly associated with L2 genotypes), another one was MLST ST60, and the fourth sample, L2nv, also resulted in ST58 by MLST analysis (Table 3).

**Figure 1:**
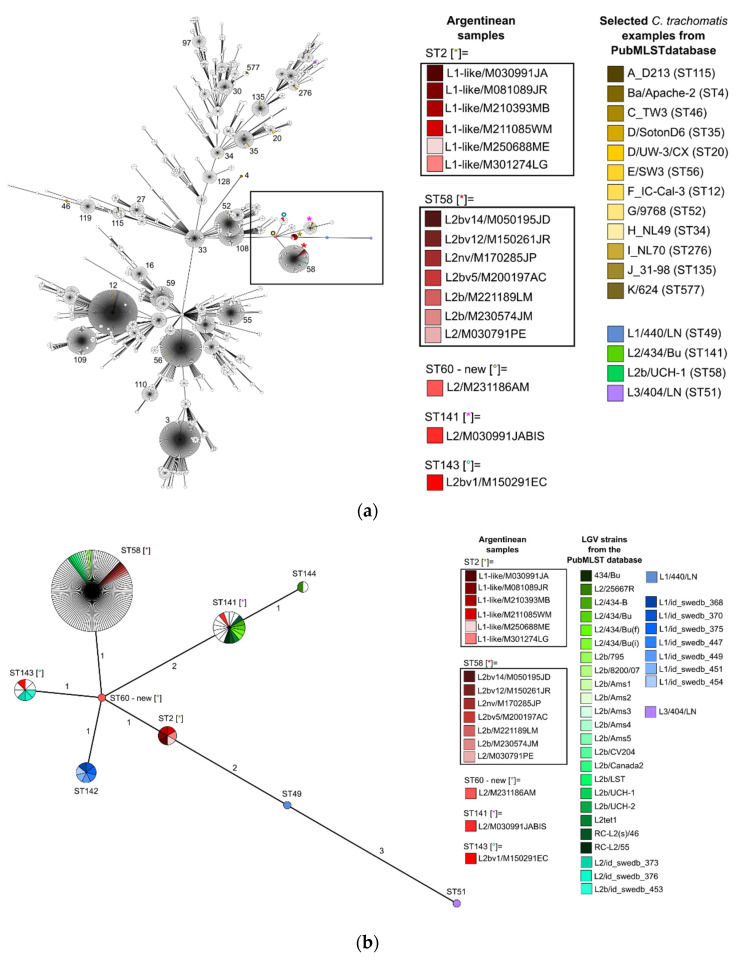
GrapeTrees showing the relationship of *C. trachomatis* STs from patients of the Buenos Aires outbreak in the context of all characterized samples published in the *Chlamydiales* PubMLST database with the Uppsala scheme. For reference: Argentinian samples are highlighted in shades of red, *C. trachomatis* examples from the PubMLST database are referenced in other colors, and the remaining samples in the database that meet the selection criteria remain white. Different colored asterisks and small circles indicated in square brackets within the figure correspond to the different STs of the Argentinean samples as shown in the legend of the graph. (**a**) GrapeTree clustering of 560 STs available in the database containing 3402 samples. The black box indicates where Argentinean samples are located. The most common STs are indicated by number; (**b**) GrapeTree clustering of all (n = 160) available LGV strains and their available STs (https://pubmlst.org/bigsdb?db=pubmlst_chlamydiales_isolates (accessed on 24 November 2023)). Sphere size is proportional to the number of samples of each sequence type. The numbers on the branches represent the number of alleles that varies between STs (single- or double-locus variants).

To contextualize the relationship of *C. trachomatis* STs from the Buenos Aires outbreak, we performed two GrapeTree analyses using data from the PubMLST database (Figure 1). Of the 560 STs associated with *C. trachomatis* Uppsala scheme, we identified 9 STs (ST = 2, 49, 51, 58, 60, 141, 142, 143, and 144) associated with LGV based on sequences and epidemiological information. Our samples were associated with STs related with L2 and L2b (ST = 58, 141, and 143), a newly associated ST2 linked to the L1-like, and a novel ST60 (*omp*A = L2), centrally positioned in the diagram (Figure 1b), connecting with all other STs.

### 3.3. Comparative Analysis of Sequence Types by MLST and Clinical Symptoms

An analysis of the patient’s clinical manifestations and STs was performed (Table 4). This analysis focused on the subset of 16 patients for whom MLST results were available, enabling a comparison with clinical symptomatology. Symptoms in this subset of patients were diverse. Notably, fistula formation was consistently absent in all cases. Most cases presented with rectal syndrome (14 patients). ST58 was associated with other STIs besides HIV (*p* = 0.0291). The frequency of HIV infection was higher in patients with *C. trachomatis* ST58, and having an inflammatory tumor was more frequent in patients with ST2, but no statistically significant association was reached. Concerning sexual behavior, 14 patients declared to have active and passive anal sexual practices and oral–anal intercourse, 1 patient claimed passive practices, and another passive practice and oral–anal intercourse. All of them were MSM.

## 4. Discussion

This study provides significant contributions to understanding the genetic diversity of *C. trachomatis* LGV strains in Argentina, filling a notable gap in the data not only within Argentina but also across South America. It represents the first comprehensive investigation of this issue in the region. In addition to *omp*A gene sequencing, our study employed Multilocus Sequence Typing (MLST), offering a more comprehensive approach. However, a limitation of this study is the absence of complete genome analysis from the samples, which could have provided additional insights. Our results reveal a substantial variability in the *omp*A gene sequences of the L2b strains, which, in contrast to the diversity observed in the European outbreak, is notable for occurring within a relatively short timeframe and limited geographical area. It is important to note that the European variability stems from studies conducted over several years, encompassing diverse geographical regions and a large number of samples [12,27,33,34,35]. We identified three novel variants, designated as L2bv12, L2bv13, and L2bv14, and one strain with a mutation AM884176.1: g.59122A>T that defies classification as either L2 or L2b. For this strain, named as L2nv, we propose the designation L2i for this variant according to Helena M. B. Seth-Smith et al. (2021) [26,27]. Given the complexity and lack of consensus surrounding the nomenclature of *Chlamydia trachomatis* strains, coupled with the current challenge of distinguishing between closely related variants, we advocate for a systematic approach in assigning such designations, based on the work of Helena M. B. Seth-Smith et al., 2021 [27]. As the *omp*A of L2b was defined while being 1bp different from the *omp*A of L2, we suggest that strains exhibiting the mutation characterizing L2b in the *omp*A gene (A485G) be classified as variants of L2b (L2bv12, L2bv13, and L2bv14). Conversely, strains lacking the L2b mutation could be assigned a new letter designation (L2i) to denote their distinct identity.

The sequences of strains initially labeled as L1 exhibited remarkable homogeneity and differed significantly from the standard L1. Therefore, we provisionally designate them as L1-like pending further detailed analysis. These strains were assigned to ST2, which, as of our study, lacked uploaded sequences and associated epidemiological characteristics. It is noteworthy that we have had a high proportion of L1-like strains since 2017, indicating a unique epidemiological trend not previously documented in South America and sporadically globally reported [27]. All our L1-like samples exhibited up to 10 mutations compared to L1/440 in the *omp*A gene. Nine of these mutations had been previously documented in a strain from a patient in the United States diagnosed in the early 1980s (GQ413956.1) that was published in a 2010 study employing MLST on strains isolated from MSM in Europe and the United States. Furthermore, compared to the strain found in the US patient, our study strains exhibit differences in two of five alleles, as determined by the MLST Uppsala scheme (CT144 and *pbp*B) [36]. These findings highlight the emergence of novel *omp*A genotypes within the circulating LGV strains in Buenos Aires, Argentina, with an epidemiological pattern different from Europe and the US [33,37,38,39]. The discovery of the L1-like strain raises questions about its appearance and evolution; thus, a study of complete genomes would provide a better understanding.

In addition, a new sequence type, ST60, was identified within the L2 genotype. Despite the homogeneity of L2 based on *omp*A sequences, it exhibited three different STs, with each sample analyzed representing a unique ST, highlighting significant variability within the strains denoted as the L2 type. Furthermore, the variant analysis conducted by Seth-Smith et al. focused on the evolution of *C. trachomatis* lymphogranuloma venereum from the European outbreak, revealing a common reversion to the *omp*A genotype L2 with the L2b genomic backbone [27,40]. In contrast to our results, all genomes analyzed in that study turned out to be ST58 according to the Uppsala scheme of *C. trachomatis*. Therefore, there is a possibility that our samples, classified as L2 according to the *omp*A gene study and MLST ST58, also exhibit an L2b genomic backbone. However, this remains uncertain without the analysis of complete genomes [41,42,43,44].

In previous studies, we have compared LGV and non-LGV genotypes in symptomatic patients in Buenos Aires. Among patients presenting with anorectal symptoms, LGV genotypes were found to predominate over non-LGV genotypes. While clinical manifestations are not pathognomonic of a specific biovar, notable differences were observed between the two groups. Specifically, older age and HIV-positive status were significantly higher in the LGV group. Anal discharge, bleeding, severe proctitis, and anal ulcers were more commonly reported in the LGV group [9]. In this study, we delved deeper into the LGV group and utilized MLST data to conduct a comparative analysis of sequence types (STs) with associated symptoms. Our analysis revealed that, despite the small number of samples studied, it was possible to demonstrate the association of ST58 with other STIs besides HIV. The confirmation of the differences in clinical manifestation for ST2 (the presence of an inflammatory tumor) and the correlation between a greater frequency of HIV and ST58 can be supported by studying a larger set of samples.

The prevalence of LGV was high in our study, probably because it was a population exhibiting symptoms associated with proctitis. At the same time, the modest sample size limits the conclusions drawn from our results. For example, among LGV-infected patients, there was a higher frequency of HIV-infected individuals than the overall HIV prevalence in the study population, or even the HIV prevalence in *C. trachomatis*-infected patients, but no statistical significance was demonstrated.

In summary, our findings not only reveal the presence of previously unreported genotypes but also emphasize the evolving landscape of LGV strains in Buenos Aires. The absence of L1-like strains in global databases and their emergence in our region points out the importance of continued genomic and epidemiologic surveillance, highlighting the unique dynamics of *C. trachomatis* genotypes in different geographical contexts [12,13,38,45]. Within the L2b strains, we identified three novel *omp*A L2b variants. Additionally, a new *omp*A L2i variant was proposed, adding to the observed variability. The discovery of a novel sequence type (ST60) within the L2 genotype further contributes to the genetic diversity uncovered in our study. Furthermore, despite the relatively small sample size, our study revealed a noteworthy diversity of sequence types within the L2 group. Lastly, ST58 was associated with the concomitant presence of another STI in addition to HIV. All of these findings underscore the importance of further investigation into the genomic intricacies of *C. trachomatis* strains and their comparison against the epidemiological characteristics of the patients in our region.

## Figures and Tables

**Table 1:** Variability of the *omp*A gene in *Chlamydia trachomatis* LGV L2b.

Samples (N)	Percentage (L2b = 38)	Mutated Bases	*omp*A-Genotype Designation ^a^	DNA Characterization	Protein Characterization	Mutation Type	Protein Mutation Site
29	76.32%	0	L2b	100% identical to JN795427.1 ^b^	No variation	-	-
4	10.53%	1	L2bv1	JN795427.1:c.517C>A	JN795427.1:p.(Leu151Ile)	Conservative	VD2 ^c^
1	2.63%	2	L2bv5	JN795427.1:c.271G>A JN795427.1:c.493C>A	JN795427.1:p.(Ala69Thr) JN795427.1:p.(His143Asn)	Conservative Conservative	VD1 VD2
1	2.63%	1	L2bv11	JN795427.1:c.1000G>A	JN795427.1:p.(Ala312Thr)	Conservative	VD4
1	2.63%	1	L2bv12 (New)	JN795427.1:c.490A>G	JN795427.1:p.(Asn142Asp)	Conservative	VD2
1	2.63%	1	L2bv13 (New)	JN795427.1:c.802G>A	JN795427.1:p.(Asp246Asn)	Conservative	Loop5
1	2.63%	2	L2bv14 (New)	JN795427.1:c.494A>G JN795427.1:c.515A>C	JN795427.1:p.(His143Arg) JN795427.1:p.(Lys150Thr)	Conservative Semi-conservative	VD2 VD2

^a^ Designations used in Helena M. B. Seth-Smith et al., 2021 (Microb Genom. 2021 Jun;7(6):000599) [27]. ^b^ L2b reference sequence, GenBank JN795427.1: *C. trachomatis* serovar L2b main outer membrane protein gene, partial CDS. ^c^ VD: Variable domain (region).

**Table 2:** Allelic profiles of sequence types (STs) according to the Uppsala scheme and sample distribution.

ST	CT058	CT144	CT172	hctB	pbpB	Samples (n)
2	13	4	13	18	28	6
58	13	17	13	27	28	7
60 (new)	13	17	13	18	28	1
141	13	19	6	18	28	1
143	13	17	13	44	28	1

**Table 3:** MLST-derived STs and *omp*A genotypes.

Sample	ST	*omp*A Genotype
M030991JA	ST2	L1-like
M211085WM	ST2	L1-like
M081089JR	ST2	L1-like
M301274LG	ST2	L1-like
M250688ME	ST2	L1-like
M210393MB	ST2	L1-like
M030791PE	ST58	L2
M170285JP	ST58	L2nv
M230574JM	ST58	L2b
M221189LM	ST58	L2b
M200197AC	ST58	L2bv5
M150261JR	ST58	L2bv12
M050195JD	ST58	L2bv14
M231186AM	ST60 (New)	L2
M030991JAB	ST141	L2
M150291EC	ST143	L2bv1

The table: Summary of MLST results and *omp*A genotypes. This table presents data from 16/20 samples from which MLST results were successfully obtained. The table consists of three columns: sample (encoded sample ID), ST (MLST result), and *omp*A genotype. To assign *omp*A genotypes to the samples, sequences obtained via Sanger sequencing were compared with the NHI database using the BLAST tool and, more specifically, L2b subtypes were designated based on the study by Helena M. B. Seth-Smith et al., 2021 [27].

**Table 4:** Comparison of STs (sequence types by MLST) with patient symptomatology.

Sample	ST	Proctalgia	Anal Ulcer	Severity of Proctitis	Inguinal Lymphadenopathy	Tumor	HIV	Other STIs ^b^
M030991JA	ST2	−	−	Moderate	+	Anorectal	+	GC/HPV/Sy
M211085WM	ST2	+	−	Not verified ^a^	−	−	−	−
M081089JR	ST2	+	−	−	−	Anorectal	+	−
M301274LG	ST2	+	−	Severe	+	−	−	−
M250688ME	ST2	+	+	Mild	+	Anorectal	+	−
M210393MB	ST2	−	−	Moderate	+	Anorectal	+	−
M030791PE	ST58	−	+	Severe	+	−	+	HPV/Sy
M170285JP	ST58	+	−	Moderate	+	−	+	HSV/HPV
M230574JM	ST58	−	+	Severe	−	−	+	−
M221189LM	ST58	+	−	Moderate	+	−	+	GC
M200197AC	ST58	−	−	Mild	+	Anorectal	+	GC/Sy/HSV
M150261JR	ST58	−	−	Moderate	−	−	+	GC/HPV
M050195JD	ST58	+	−	Moderate	−	−	+	Sy
M231186AM	ST60	+	+	Severe	+	Anorectal	+	GC
M030991JAB	ST141	−	−	Moderate	+	Anorectal	+	GC/HPV/Sy
M150291EC	ST143	+	+	Severe	−	−	+	−

This table presents data from 16/20 samples from which MLST results were successfully obtained. It comprises nine columns: sample (encoded sample ID), ST (MLST result), proctalgia, anal ulcer, severity of proctitis (mild, moderate, or severe), inguinal lymphadenopathy, tumor, HIV, and other STIs. “+” indicates the presence of the symptom, while “−” indicates its absence. ^a^ Not verified, as rectoscopy was not performed. ^b^ Acronym clarification: GC (Gonorrhea), HPV (Human Papillomavirus), Sy (Syphilis), and HSV (Herpes Simplex Virus).

## Data Availability

All the data presented are available in PubMLST database (https://pubmlst.org/, Acc. No. 5059-5074) and in GenBank (https://www.ncbi.nlm.nih.gov/genbank/, Acc. No. MN548736.1 to MN548759.1, MN537150.1 to MN537152.1, and MN563608.1 to MN563615.1, accessed on 24 November 2023). The study was approved by an Ethics Committee (“Detección de *C. trachomatis* en pacientes con rectitis infecciosa: prevalencia y tipificación”, Gobierno de la Ciudad de Buenos Aires, No. 201723) and patients provided written informed consent.

## Supplementary Materials

The following supporting information can be downloaded at: https://www.mdpi.com/article/10.3390/microorganisms12030587/s1, Table S1: Summary of Patient Characteristics, including Gender, Sexual Behavior, Disease Type (HIV+/HIV−), *omp*A Genotype, *omp*A Mutations, and MLST Results.
